# Complete Genome Sequences of *Pseudomonas fluorescens* Bacteriophages Isolated from Freshwater Samples in Omaha, Nebraska

**DOI:** 10.1128/genomeA.01501-16

**Published:** 2017-03-23

**Authors:** Guoqing Lu, Jamie Luhr, Andrew Stoecklein, Paige Warner, William Tapprich

**Affiliations:** Department of Biology, University of Nebraska at Omaha, Omaha, Nebraska, USA

## Abstract

The complete genome sequences of four *Pseudomonas fluorescens* bacteriophages, UNO-SLW1 to UNO-SLW4, isolated from freshwater samples, are 39,092 to 39,215 bp long. The genomes are highly similar (identity, >0.995) but dissimilar from that of *Pseudomonas* phage Pf-10 (the closest relative, 0.685 to 0.686 identity), with 48 to 49 protein-coding genes and 66 regulatory sites predicted.

## GENOME ANNOUNCEMENT

*Pseudomonas fluorescens* is a common Gram-negative motile rod-shaped bacterium with features that make it environmentally, commercially, and clinically relevant (1). For example, it is responsible for milk spoilage (2), and infection by *P. fluorescens* is correlated to Crohn’s disease (3). Here, we report the genome sequences of four bacteriophages (UNO-SLW1 to UNO-SLW4) that infect *P. fluorescens* Migula strain (ATCC 27663), isolated from water samples collected in and around Omaha, NE, USA. Phage genomic libraries were constructed with the Nextera DNA library preparation kits and sequenced on an Illumina HiSeq 2500 sequencer. A total of 70.1 million reads (100 bp each), ranging from 11.6 million in UNO-SLW1 to 22.1 million in UNO-SLW2, were generated. Sequences were assembled using Velvet 1.2.10 (4) on the Galaxy Queensland server (http://galaxy-qld.genome.edu.au/galaxy/). The assembled genome was annotated using the Rapid Annotations using Subsystems Technology (RAST) server (5), PHage *in silico* Regulatory Elements (PHIRE 1.0) (6), and ARNold (7).

The assembled genome lengths of *Pseudomonas* phages UNO-SLW1 to UNO-SLW4 were 39,215, 39,167, 39,092, and 39,136 bp, with 1,350×, 1,395×, 1,701×, and 1,018× coverage, respectively. The G+C content of the genomes was approximately 57.9%. The pairwise sequence identities among the four genomes ranged from 0.996 to 0.997. The aligned genome sequences are 39,275 bp long, where an insertion of 93 bp from positions 8807 to 8900 and a deletion of 46 bp from positions 20732 to 20777 were found in SLW1, an insertion of 103 bp from positions 20690 to 20793 was found in SLW2, an insertion of 23 bp from positions 9257 to 9270 and a deletion of 87 bp from positions 20690 to 20777 were found in SLW3, and a deletion of 30 bp from positions 20763 to 20793 were found in SLW4. The top BLAST hit of the whole-genome sequences is *Pseudomonas* phage Pf-10 (accession no. KP025626.1). The identities between the genomes of *Pseudomonas* phage Pf-10 and the four *Pseudomonas* UNO phages were all <0.70, indicating that the UNO phages are new and have not been reported previously (8). Electron microscopy and genome analysis suggest the UNO phages are T7-like bacteriophages (Podoviridae). As with the closest BLAST hit (*Pseudomonas* phage Pf-10), terminal direct repeats were not detected in our phage genomes.

A total of 48 to 49 proteins were predicted in the UNO phage genomes, including 27 that can be functionally annotated and 21 to 22 that are hypothetical proteins. The predicted proteins with known function are indicative of T7-like phages. These include T7-like phage single-stranded DNA (ssDNA)-binding protein, T7-like phage primase or helicase protein, T7-like phage exonuclease (EC 3.1.11.3), T7-like phage endonuclease (EC 3.1.21.2), T7-like phage DNA polymerase (EC 2.7.7.7), T7-like tail tubular proteins A and B, T7-like phage head-to-tail joining protein, and DNA-directed RNA polymerase (EC 2.7.7.6). A total of 66 regulatory sequences were predicted, including ribosome binding sites for all genes, 11 phage-specific promoters, four host-specific promoters, and three rho-independent terminators.

### Accession number(s).

The complete genome sequences of the four *Pseudomonas* phages, UNO-SLW1, UNO-SLW2, UNO-SLW3, and UNO-SLW4, have been deposited in the NCBI GenBank under accession numbers KX431888, KX449361, KX449362, and KX449363, respectively.
